# Performance of a Salt Check Sheet for Screening Salt Intake Estimated From 24-hour Urinary Sodium Excretion in Middle-aged Japanese Adults Following a Salt Reduction Intervention

**DOI:** 10.2188/jea.JE20240493

**Published:** 2026-01-05

**Authors:** Sachiko Maruya, Shiori Sugawara, Mayuka Matsumoto, Misako Nakadate, Junko Ishihara, Ribeka Takachi

**Affiliations:** 1 Department of Food Science and Nutrition, Nara Women’s University, Nara, Japan; 2 Department of Health and Nutrition, Nagoya Aoi University, Nagoya, Japan; 3 Department of Health and Nutrition, Sendai Shirayuri Women’s College, Sendai, Japan; 4 Department of Food and Life Science, Azabu University, Kanagawa, Japan

**Keywords:** salt, ROC, urinary excretion, validation study, reduced salt diet

## Abstract

**Background:**

One of the factors for not achieving a reduced salt diet may be the difficulties in screening individuals according to their quantitative salt consumption. Accordingly, we examined the performance of a simplified 13-item salt check sheet as a quantitative tool for screening excessive salt intake by comparing with the salt intake amount measured by 24-hour urinary sodium (salt equivalent g/day) excretion.

**Methods:**

One hundred fifty-four participants (57 males and 97 females) from Kanagawa, Tokyo, and Nara Prefectures in Japan were included. In this study, which the design is a cross-sectional validation study, the salt intake amount was used as a diagnostic criterion, and corresponding receiver operating characteristic (ROC) curves were prepared based on the sensitivity and specificity of each score of the salt check sheet.

**Results:**

The average salt intake were 13.5 and 10.2 g/day for males and females, respectively. When using the total score, among males, the area under the ROC curve (AUC) was moderate (0.702; 95% confidence interval [CI], 0.543–0.862), confirming its value as a diagnostic tool for salt intake of ≥10 g/day. In females, the AUCs were low for any criteria. When score calculation item was limited to three that contributed to the higher salt intake in this population, the AUC for ≥10 g salt/day was moderate (0.700; 95% CI, 0.595–0.805).

**Conclusion:**

The salt check sheet was found to be useful in screening for excessive salt intake only in males. For females, it was suggested that it could be used only when three specific items are used.

## INTRODUCTION

Excess salt or salted food intake is a risk factor for cardiovascular diseases and gastric cancer.^1^^,^^2^ High intake of salt is the leading dietary risk factor for deaths and disability-adjusted life-years globally.^3^ Reducing salt intake is one of the priority actions for addressing the crisis of noncommunicable diseases in this decade.^4^ However, World Health Organization’s recommendation of <5 g salt intake per day has not been achieved worldwide to date.^5^^,^^6^

One of the factors for not achieving a reduced salt diet may be the difficulties in screening individuals according to their quantitative salt consumption, albeit there is also a need for clinical and nutritional education which focuses on a reduced salt diet. Multiple 24-hour urine excretion is recognized as a reliable method for evaluating actual salt consumption, but it is not a feasible and versatile screening method because of the heavy burden in urine collection.^7^

Food frequency questionnaires (FFQs), comprising a substantial number of foods, including salty foods, as well as salt-related behaviors, and have been widely used in epidemiological studies to evaluate the habitual intake of nutrients or foods by ranking the individuals within a study population and the validity of the ranking has been reported for this purpose.^8^ However, including many questions can still be burdensome among respondents. Wakai et al reported that when comparing the validity between short (<70 items) and long (≥97 items) FFQs for estimating nutrient intake, a slight difference was observed (for sodium or salt, *r* = 0.33, and 0.32, respectively).^9^ Thus, it is reasonable to use a short and simple questionnaire in dietary assessment. Tsuchihashi et al have developed a short and simplified 13-item questionnaire that is specific for testing salt intake amount with little burden to respond (salt check sheet), and those items reflect the characteristics of the Japanese diet (See also Table 1).^10^^,^^11^ To date, this questionnaire has been validated only for the purpose of ranking individuals by correlation coefficient.^10^^,^^11^ The performance screening for assessing quantitative salt intake amount for clinical use has not been examined to date.

**Table 1.** Characteristics of the study participants (*n* = 154; middle-aged Japanese adults)

	Males	Females
Number^a^	57	97
Age, years^b^	44.0 (10.5)	47. 9 (8.8)
Age group, years^c^		
20s	8 (14.0)	2 (2.1)
30s	8 (14.0)	12 (12.4)
40s	21 (36.8)	41 (42.3)
50s	17 (29.8)	33 (34.0)
≥60s	3 (5.3)	9 (9.3)
Height, cm^b^	172.6 (5.2)	158.0 (5.2)
Body weight, kg^b^	70.8 (10.4)	54.7 (9.5)
BMI, kg/m^2 b^	23.8 (3.2)	21.8 (3.3)
Participants with BMI ≥25 kg/m^2 c^	18 (31.6)	15 (15.5)
Participants on medication^c^	9 (15.8)	27 (27.8)
24-hour urinary sodium excretion, mg/day^d,e^	4,563 (1,957, 1,925–12,575)	3,456 (1,227, 1,360–8,177)
Salt intake, g/day^d,e^	13.5 (5.8, 5.7–37.1)	10.2 (3.6, 4.0–24.1)
Total salt check sheet score, points^d,e^	14.1 (4.5, 6–26)	11.4 (4.3, 4–21)

BMI, body mass index.

^a^Who did not take antihypertensive drug at the baseline of the randomized controlled trial.

^b^Mean (standard deviation).

^c^Number (%).

^d^Salt intake was obtained by 24-hour urinary sodium excretion using the following formula: 24-hour urinary excretion of sodium (mg/day) × 2.54/1,000/0.86.

^e^Mean (standard deviation, minimum–maximum).

In the present study, we aimed to examine the performance of using the simplified salt check sheet as a quantitative diagnostic tool by comparing its results with the amount of salt intake measured using 24-hour urinary excretion of sodium.

## METHODS

### Study setting and participants

The participants in this study were recruited from a previous study titled “A randomized controlled trial presenting a reproducible reduction salt method,” which was jointly conducted by Sagami Women’s University, Azabu University, and Nara Women’s University.^12^ That was a randomized controlled trial aiming to examine whether the effects of visual monitoring of salt concentration in homemade dishes and the use of low-sodium seasoning (eg, miso and soy sauce, prepared from soy beans) on reducing salt intake among Japanese people. The study was conducted between June 2015 and March 2018. The inclusion criteria were males and females aged >20 years in Kanagawa, Tokyo, and Nara Prefectures who were not taking antihypertensive drugs and did not have soy allergies. There were 195 participants in total. In the final follow-up survey of the trial at 9 months after the intervention ended, (ie, 12 months after the baseline survey conducted between July 2016 and March 2018), a 24-hour urine collection and the salt check sheet were conducted simultaneously. Finally, 154 participants (57 males and 97 females) who completed both and met all inclusion criteria (eg, no antihypertensive drug use at baseline) were included in the analysis. From the trial, we reported that short-term reduction of urinary sodium excretion via salt restriction of home dishes returned to the baseline level after 12 months after the baseline for all groups.^12^

This study was registered with the UMIN-CTR clinical trial registration system (UMIN000017773) and conducted according to the guidelines laid down in the Declaration of Helsinki. All procedures involving research study participants were approved by the Ethics Review Boards, with full reviews, of Sagami Women’s University (approval date: May 12, 2015, ethical code: 14135), Azabu University (approval date: April 17, 2017), and Nara Women’s University (approval date: December 07, 2015). Written informed consent was obtained from all participants.

### Salt check sheet

The simplified questionnaire, the salt check sheet, comprises only 13 items, and the scoring system was developed by Tsuchihashi et al to conveniently evaluate the salt intake of individuals at sites such as health check-ups, and the details have been reported previously (see also Table 1).^10^^,^^11^

The salt check sheets were distributed at the study orientation or mailed to the participants. All participants were then invited to the study venue where they submitted their completed salt check sheets. Each answer was converted to 0 to 3 points; then, the answers were summed up to a total score of 35 points.^10^^,^^11^ A higher score means higher salt intake. All participants were also asked to provide their height and weight according to self-report.

### Measurements

Urine samples were collected using the Sumius U-Container, a Precise Urine Measurement device (Sumitomo Bakelite Co, Ltd, Tokyo, Japan), which can obtain a 1/50 portion of all collected urine samples over 24 hours.^13^ We distributed the device and urine volume record sheet either by mail or during the study orientation. Participants were also asked to record the start and end dates, menses, urine volumes (from the second urination of the day until the first urination the next morning), and any error in the collection of a sample. We obtained the specimen with the device kept in a cold dark place and sent to a laboratory within 2 days. Urinary sodium concentrations (mEq/L) were analyzed at Koto Microbial Research Institute, Inc., using electrode potentiometry. Individuals who reported two or more urine collection errors (eg, missed sample collections or sample spills) were excluded from the analysis. The 24-hour urinary sodium excretion of sodium was calculated using the following formula: 24-hour urinary sodium excretion (mg/day) = obtained excretion volume (mL) × 50 × urinary sodium concentration (mEq/L) × 23/1,000. Single-time error was corrected by their average excretion volume of each urination. Moreover, urinary sodium excretion was converted and adjusted to salt intake amount (g/day) using the following formula: 24-hour urinary sodium excretion (mg/day) × 2.54/1,000/0.86.^14^ The data of 14 participants were corrected owing to one missed urine collection.^15^

### Statistical analysis

Spearman’s correlation coefficient was calculated to compare the total score obtained with the salt check sheet with the salt intake amount.

Diagnostic criteria values were between 6–12 g in 1 g increments. In Japan, the recommended daily salt intake for managing hypertension is 6 g and for the population in East Asia, the average daily intake is 12 g.^16^^,^^17^ Therefore, we chose these values as the upper and lower values in the diagnostic criteria range. For each sex and the above diagnostic criteria value, the sensitivity and specificity of each salt check sheet total score was calculated, and a receiver operating characteristic (ROC) curve was prepared from the sensitivity and false positive rates (1 − specificity); then, the area under the ROC curve (AUC) was obtained. The ROC curves were judged to be moderately useful with AUC of ≥0.7.^18^^,^^19^ Further, to determine the optimal cutoff value, the positive predictive value, Youden’s Index (sensitivity + specificity − 1), and distance between each point on the ROC curve and the upper left hand of the corner (0, 1) point were examined. For the sensitivity, specificity and Youden’s Index, higher values were judged to be better. Contrarily, a lower value for the distance from the (0, 1) point was judged to be better. Finally, the optimal cutoff value was determined by considering the balance of these indices.^18^^,^^19^

To improve the diagnosis based on the score in cases of low correlation using the total salt check sheet score, multiple regression analysis was conducted using the score of each item on the salt check sheet (points) as the independent variable with salt intake amount (g/day) as the dependent variable. Alternative scores based on highly contributed items in the salt check sheet to salt intake amount were similarly analyzed via Spearman’s correlation coefficient and analysis using the ROC curves. All statistical analyses were performed according to sex using SPSS software (versions 21.0, 25.0, and 28.0; IBM, Inc., Armonk, NY, USA). Significance level was set at *P* < 0.05.

## RESULTS

The characteristics of all 154 participants (57 males and 97 females) are shown in Table 1. The average ages were 44.0 (standard deviation [SD], 10.5) and 47.9 (SD, 8.8) years for males and females, respectively (range, 21–70 years). The proportions of those with a body mass index (BMI) ≥25 kg/m^2^ were 31.6% and 15.5% for males and females, respectively. The average (SD, minimum-maximum) amount of 24-hour urinary sodium excretion and total score obtained from the salt check sheet were 4,563 (SD, 1,957; range, 1,925–12,575) mg/day and 14.1 (SD, 4.5, range, 6–26) points, respectively, for males and 3,456 (SD, 1,227; range, 1,360–8,177) mg/day and 11.4 (SD, 4.3; range, 4–21) points, respectively, for females. These 24-hour urinary sodium excretion values corresponded to a salt intake amount of 13.5 (SD, 5.8; range, 5.7–37.1) g/day for males and 10.2 (SD, 3.6; range, 4.0–24.1) g/day for females (Table 1).

A positive weak significant correlation between the total salt check sheet score and salt intake amount was found for males (*r* = 0.318, *P* = 0.02), whereas for females, this was weak and not significant (*r* = 0.183, *P* = 0.07) (data not shown). Considering participants’ prior exposure to salt reduction interventions, stratified analyses were performed according to the original randomized controlled trial methods: either (1) monitoring-based intervention or (2) low-sodium seasoning use.^12^ No systematic differences in correlation coefficients were observed between males and females depending on the presence or absence of the monitoring or low-sodium seasoning intervention (monitoring intervention: *r* = 0.327 and 0.174, respectively, for males, and *r* = 0.145 and 0.239, respectively, for females; low-sodium seasoning intervention: *r* = 0.224 and 0.296, respectively, for males, and *r* = 0.162 and 0.196, respectively, for females) (data not shown).

Furthermore, we performed additional sensitivity analyses excluding participants on medication at the time of present study. The analyses revealed that the correlation coefficient between the total score of the salt check sheet and salt intake slightly improved for both sexes (*r* = 0.355, *P* = 0.01 for 48 males, *r* = 0.231, *P* = 0.05 for 70 females, respectively) (data not shown).

Moreover, considering the participants’ wide age range, we conducted age-stratified analyses (younger group: 21–49 years; older group: 50–70 years). No significant changes occurred by age group (data not shown).

**Figure 1.**
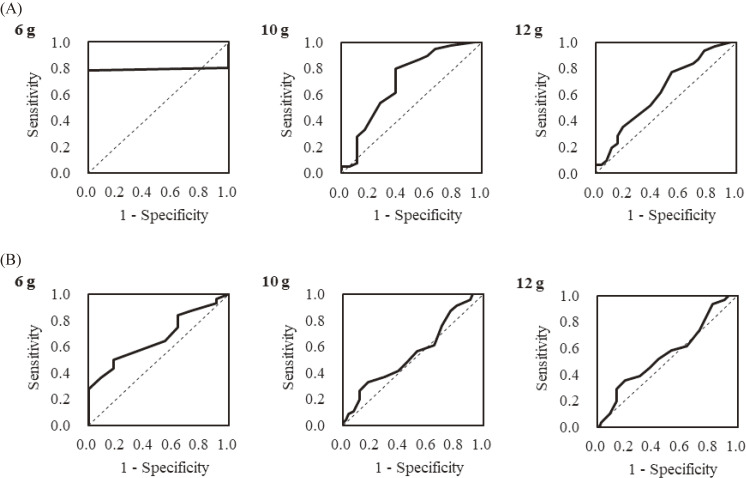
ROC curves based on the total score of the salt check sheet for each criterion (6, 10 and 12 g salt intake measured by 24-hour urinary excretion): (**A**) men and (**B**) women. ROC, receiver operating characteristics.

The AUC values for males and females are shown in Table 2, and the ROC curve for the criteria on salt intake amount of 6 (low), 10 (average on Japanese) and 12 g (high) are presented in Figure 1. In males, for the criteria of 6 and 10 g salt intake amount, the AUCs were higher than 0.7. For the detection of 6 g salt intake, the optimal cutoff point could not be determined because only one participant had a salt intake amount of <6 g. As a result, the optimal cutoff value for detection of ≥10 g salt intake was determined to be a total salt check sheet score of 13 points (Table 3).

**Table 2.** AUC and 95% CI based on the total salt check sheet score according to each criterion for salt intake amount (expressed as gram per day) measured by 24-hour urinary excretion among males and females (*n* = 154)

Males (*n* = 57)			Females (*n* = 97)		

Criterion	Over/Under Criteria (*n*)	AUC (95% CI)	Criterion	Over/Under Criteria (*n*)	AUC (95% CI)
≥6 g	56/1	0.795 (0.688–0.901)	≥6 g	86/11	0.666 (0.523–0.809)
≥7 g	51/6	0.631 (0.364–0.897)	≥7 g	79/18	0.592 (0.455–0.730)
≥8 g	48/9	0.565 (0.341–0.788)	≥8 g	66/31	0.611 (0.491–0.731)
≥9 g	44/13	0.610 (0.406–0.775)	≥9 g	57/40	0.610 (0.496–0.724)
≥10 g	39/18	0.702 (0.543–0.862)	≥10 g	46/51	0.557 (0.442–0.672)
≥11 g	35/22	0.681 (0.535–0.826)	≥11 g	36/61	0.567 (0.442–0.672)
≥12 g	31/26	0.629 (0.482–0.776)	≥12 g	31/66	0.559 (0.442–0.672)

AUC, area under the receiver operating characteristic curve; CI, confidence interval.

**Table 3.** Indices for each cutoff value according to the total salt check sheet score for 10 g salt intake measured by 24-hour urinary excretion among males (*n* = 57)

Cutoff (total score)	Sensitivity	Specificity	Positive predictive value	Youden’s Index	Distance to the (0, 1) point
≥7	1.000	0.056	0.696	0.056	0.892
≥8	0.974	0.222	0.731	0.197	0.606
≥9	0.949	0.333	0.755	0.282	0.447
≥10	0.897	0.389	0.761	0.286	0.384
≥11	0.897	0.389	0.761	0.286	0.384
≥12	0.872	0.444	0.773	0.316	0.325
**≥13**	**0.795**	**0.611**	**0.816**	**0.406**	**0.193**
≥14	0.615	0.611	0.774	0.226	0.299
≥15	0.538	0.722	0.808	0.261	0.290
≥16	0.333	0.833	0.813	0.167	0.472
≥17	0.282	0.889	0.846	0.171	0.528
≥18	0.231	0.889	0.818	0.120	0.604
≥19	0.179	0.889	0.778	0.068	0.686
≥20	0.179	0.889	0.778	0.068	0.686
≥21	0.077	0.889	0.600	−0.034	0.864
≥22	0.051	0.944	0.667	−0.004	0.903
≥23	0.051	1.000	1.000	0.051	0.900

**Figure 2.**
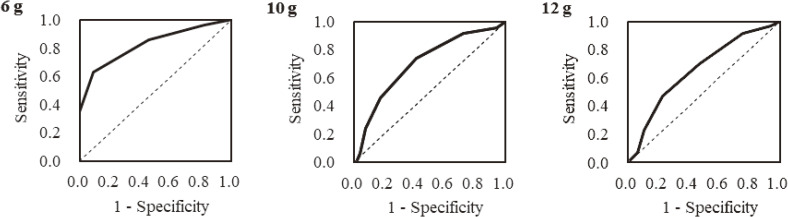
ROC curves based on the salt check sheet score using three items of the sheet for each criterion (6, 10, and 12 g salt intake measured by 24-hour urinary excretion) among women. ROC, receiver operating characteristics.

For females, the AUCs were <0.7 for any criteria, and their usefulness were considered to be low and determined not to be useful. Thus, we performed multiple regression analysis to examine which item contributed highly to the salt intake amount in females. The results of the multiple regression analysis are shown in Table 4, demonstrating that the scores of following three items relatively and highly contributed to the salt intake amount in between-person difference: “Frequency of eating miso soup, soup, and so on,” “How much soup in noodles, such as udon, ramen, or others, do you consume?” and “Do you think you eat a lot?”. Although the contribution of item “Frequency of eating miso soup, soup, and so on” to salt intake amount was clearly not significant in this study, we adopted it because of its high contribution to Japanese salt intake.^20^^,^^21^ When using these three items, the full score was 9 points. As a result, we observed a significant correlation between salt intake and the scores derived from the abovementioned three items among females (*r* = 0.440, *P* < 0.001) (data not shown; see also Figure 2, Table 5, and Table 6).

**Table 4.** Relationship between variables in the salt check sheet and salt intake amount according to multiple linear regression analyses among Females (*n* = 97)

Independent variable	Non-standardized coefficient		Standardized partial regression coefficient	*P* value

	Partial regression coefficient	SE		
**Frequency of eating miso soup, soup, and so on.**	**0.703**	**0.468**	**0.156**	**0.14**
Frequency of eating pickles, pickled plums, and so on.	0.095	0.453	0.021	0.84
Frequency of eating fish paste products such as *chikuwa* (tubular fish sausage) and *kamaboko* (steamed fish paste).	0.782	0.703	0.117	0.27
Frequency of eating salted and dried fish, dried fish seasoned with mirin (sweetened alcohol. for use in cooking), salted salmon, and so on.	0.750	0.671	0.114	0.27
Frequency of eating ham or sausage.	−0.087	0.520	−0.017	0.87
Frequency of eating noodles such as *udon* (Japanese wheat noodles) and *ramen* (Japanese-style Chinese noodles).	0.257	0.735	0.041	0.73
Frequency of eating *senbei* (Japanese salty crackers), *okaki* (soy sauce seasoned thinly-cut and dried rice cakes), potato chips, and so on.	−0.323	0.509	−0.069	0.53
How frequently do you season with soy sauce or other sauces?	−0.442	0.537	−0.094	0.42
**How much soup in noodles such as *udon*, *ramen*, or other soups do you consume?**	**1.257**	**0.409**	**0.325**	**<0.01**
Do you eat out or have convenience-store-bought bento (lunch plate)	−0.129	0.548	−0.029	0.82
Do you eat out or have ready-made side dishes?	−0.043	0.547	−0.009	0.94
How salty are your homemade dishes compared with those you eat out?	−0.507	0.366	−0.149	0.17
**Do you think you eat a lot?**	**0.832**	**0.431**	**0.195**	**0.06**

SE, standard error.

**Table 5.** AUC and 95% CI based on the salt check sheet score using three items of the sheet according to each criterion for salt intake amount (expressed as gram per day) measured by 24-hour urinary excretion among females (*n* = 97)

Criterion	Over/under criteria (*n*)	AUC (95% CI)
≥6 g	86/11	0.826 (0.721–0.930)
≥7 g	79/18	0.769 (0.668–0.870)
≥8 g	66/31	0.709 (0.603–0.814)
≥9 g	57/40	0.739 (0.640–0.839)
≥10 g	46/51	0.700 (0.595–0.805)
≥11 g	36/61	0.702 (0.594–0.810)
≥12 g	31/66	0.685 (0.573–0.796)

AUC, area under the receiver operating characteristic curve; CI, confidence interval.

**Table 6.** Indices for each cutoff value according to the salt check sheet score by using three items of the sheet for 10 g salt intake amount measured by 24-hour urinary excretion among females (*n* = 97)

Cutoff (3 items’score)	Sensitivity	Specificity	Positive Predictive value	Youden’s Index	Distance to the (0, 1) point
≥1	1.000	0.000	0.474	0.000	1.000
≥2	0.957	0.059	0.478	0.015	0.888
≥3	0.913	0.275	0.532	0.188	0.534
**≥4**	**0.739**	**0.588**	**0.618**	**0.327**	**0.238**
≥5	0.457	0.824	0.700	0.280	0.327
≥6	0.239	0.922	0.733	0.161	0.585
≥7	0.065	0.961	0.600	0.026	0.875
≥8	0.022	0.980	0.500	0.002	0.957

Additionally, we also conducted multiple regression analysis in males using the same method as that for females to ascertain which items contributed to salt intake. The results revealed that “Frequency of using seasoning” (β = 0.591, *P* < 0.01) and “Amount of meal consumed” (β = 0.331, *P* = 0.02) relatively and highly contributed to salt intake in males. As the same for females, we included “Frequency of eating miso soup, soup, and so on” in the male model (β = −0.135, *P* = 0.36), despite its non-significant contribution. Resultantly, the three-item composite score (maximum: 9 points) did not improve the correlation with salt intake (*r* = 0.302, *P* = 0.02) compared to the total score.

In addition, because the latest dietary reference intakes (DRIs) recommended for Japanese individuals suggest a salt intake of <7.5 g/day for males and <6.5 g/day for females to prevent noncommunicable diseases,^22^ we conducted the same analysis using these criteria. The results did not change substantially. On applying the revised DRIs for both sexes, the AUCs were <0.7 using the total score. Using three specified items with females, the AUC was 0.786 (95% CI, 0.679–0.892).

## DISCUSSION

In this study, we examined the performance of using the score of the salt check sheet for the discrimination of the criteria in absolute salt intake by comparing the results with those of the 24-hour urinary sodium excretion. A weak positive correlation was found between the salt check sheet total score and salt intake for males and a further weak correlation was found for females. When using the total score, the AUC was >0.7 among males, confirming the total score as a screening tool for salt intake of ≥10 g/day whereas the AUC was <0.7 by any criteria among females. In males, a total score of 13 points corresponded to 10 g salt intake. In females, using the total score of the salt check sheet was not useful. However, when using the specified three items, the salt check sheet may be useful because the AUC was >0.7, and a salt check sheet score of 4 corresponded to 10 g of salt intake. We demonstrated the usefulness of the simplified questionnaire, salt check sheet, to quantitatively screen excessive the salt intake for males, and we also demonstrated its possible usefulness for females when using the specific three items.

The relative validity of the total salt check sheet score by comparing it with 24-hour urinary sodium excretion has been reported by the study group who developed the salt check sheet to assess its validity. In a sample of 140 participants (23 males and 117 females), they found the total salt check sheet scores and 24-hour urinary sodium excretion values to be significantly correlated (*r* = 0.27, *P* < 0.01). A positive weak insignificant correlation coefficient was found for males (*r* = 0.30, *P* = 0.16), and a further weak correlation was found for females (*r* = 0.19, *P* = 0.04).^10^ Furthermore, prior to this, the developer group examined the relative validity of the total salt check sheet score by comparing it with salt intake predicted from the spot urine samples of 270 participants (129 males and 141 females), without distinguishing by sex, and reported a weak significant correlation (*r* = 0.30, *P* < 0.01).^11^ Our correlation coefficient analysis results for the total salt check sheet score were similar to the previous study’s findings. However, they examined the validity for only the correlation coefficient which means relative ranking. And one of these, study using 24-hour urine, was examined in population that consist mostly of females.^10^ In addition, the correlations between the salt check sheet score and either 24-hour urinary sodium excretion or salt intake predicted from urine in the present and these previous studies were weak rather than moderate.^10^^,^^11^ As a major factor, the salt check sheet assesses habitual salt intake over an extended period (approximately 1 month), whereas this and previous studies used a single time 24-hour or spot urine as a reference. Another potential factor might be the scoring system of the salt check sheet. Although the items listed seem to cover most salt intake-related items in the Japanese population, problems with the scoring system and weighting may arise.^20^^,^^21^

The ROC curve based on the total salt check sheet score was reasonable for the diagnostic criteria of 6 and 10 g salt intake measured using 24-hour urinary excretion among males, because the AUC value exceeds 0.7.^18^^,^^19^ However, for screening excessive salt intake, the 6 g diagnostic criterion may not be useful because almost all male participants consumed more than this amount. Moreover, the average salt intake among Japanese males is 10.9 g/day according to the National Health and Nutrition Survey.^23^

Contrarily, for females, no usefulness was found for any of the criteria when total scores were used. This is likely due to the weaker correlation between total score and salt intake than males, which consisted with the previous study.^10^ The sex-related differences in accuracy may be attributable to the difference in the range of salt intake (narrower intake range for females than for males: average 13.5 [SD, 5.8: range, 5.7–37.1] g/day for males and 10.2 [SD, 3.6; range, 4.0–24.1] g/day for females). Therefore, we examined the usefulness of using the three items with high contribution to salt intake among females in this population. As a result, the salt check sheet could be practical with an optimal cutoff value of 4 points by specified three items with respect to the criteria of 9 to 11 g which is approximately the average of that for Japanese females (9.3 g by weighed food record) and all Japanese population (10.1 g by weighed food record).^23^ Among these criteria, the highest sensitivity was observed for 10 g (73.9%). A high sensitivity (with relatively low specificity) accompanied by a high false-positivity is essential in this case because our goal is to alert the people to a potential of excessive salt intake. Thus, we adopted the criterion of 10 g salt intake for females.

The major strength of our study is that we examined the possibility of using a simple questionnaire to discriminate individual’s absolute salt intake using 24-hour urine sample collection as a reference standard, stratified by sex. To the best of our knowledge, only four studies have investigated the discrimination of absolute salt intake values by 24-hour urinary excretion in individuals via FFQ. Matsuno et al examined the performance of a FFQ in screening excessive salt intake (ie, ≥7.5 g for males and ≥6.5 g for females). Although they reported the moderate usefulness of five-time 24-hour urine collection for males (AUC 0.76; 95% CI, 0.56–0.95) and the moderate usefulness of 12-day weighed food record records for both males and females (AUC 0.80; 95% CI 0.62–0.98 and 0.71; 95% CI, 0.50–0.91, respectively), the FFQ had many questions (172 items), unlike the salt check sheet.^24^ Kelly et al reported the moderate usefulness of a FFQ in screening ≥100 mmol/L Na excretion in a single 24-hour urine sample collection in an Irish population (AUC 0.76; 95% CI, 0.6–0.9).^25^ However, unlike our study, they analyzed the whole population without separating males from females. The other two studies examined the screening performance of an FFQ simplified and specialized for sodium intake assessment for the detection of excessive salt intake by 24-hour urinary excretion.^26^^,^^27^ Although the AUC on each study was low (AUC 0.665) and moderate (AUC 0.713; 95% CI, 0.530–0.896), their studies were carried out on hypertension or renal patients whose disease itself or medication could influence their sodium excretion. Thus, their results may not be applicable to a healthy population. There are a few studies using methods other than 24-hour urinary sodium excretion as a reference standard to evaluate the FFQ’s excessive salt intake screening performance. Sasaki et al developed a short questionnaire focused on salt intake and examined its performance as a screening test using spot urinary sodium excretion as a reference.^28^ They reported that the AUC was 0.70 (95% CI, 0.66–0.74) when the discriminate value was ≥12.2 g salt intake for males and ≥11.4 g for females. Tsubono et al examined the performance of an FFQ comprising 141 items as a screening tool for salt intake using a 3-day weighed food records as a reference standard; and they reported a low performance of FFQ to discriminate ≥16 g salt intake (sensitivity, 48.9%; specificity, 54.6%).^29^ Given that the estimation of salt intake by spot urine excretion or weighed food record records are said to be less accurate than that by 24-hour urine, the results of those studies might somehow be unreliable.

The present study has several limitations. First, this study examined the salt check sheet, which assesses habitual daily salt intake, by comparing its results with the amount of salt intake based on single 24-hour urinary sodium excretion. Habitual salt intake might not be necessarily represented by single 24-hour urine sodium excretion, although single 24-hour urinary sodium excretion have been used widely as a reference standard in validation studies of intake estimation using FFQ.^25^^,^^27^^,^^30^ If the 24-hour urinary sodium excretion measurement could be repeated more than once for each participant, the precision might be increased.^31^ Second, we used answers of the salt check sheet and urine collection from individuals who had participated in the salt reduction intervention. The intervention study showed that 24-hour urinary sodium excretion levels had returned to preintervention levels by the time the salt check sheet and urine analyzed in this study collected.^12^ The possibility that the intervention might have resulted in increased misclassifications cannot be excluded, but in fact, only 7% of the participants who had undergone the low-sodium seasoning intervention continued using low-sodium seasonings until the time of this study. Moreover, stratified analysis according to intervention experience revealed no systematic differences between males and females. Thus, we conclude that the impact of the intervention on the salt concentration of foods consumed is likely negligible. And it is noteworthy to show quantitatively how large the error is with indices, such as sensitivities. Furthermore, the generalizability of the results might be limited. Third, because the salt check sheet consists of items for assessing the dietary habits of Japanese people, our results are limited to the Japanese population. Finally, we showed that the salt check sheet may be useful for females when using the three specified items: “Frequency of eating miso soup, soup, and so on,” “How much soup in noodles, such as udon, ramen, or others, do you consume?”, and “Do you think you eat a lot?”, which were obtained by performing multiple regression analysis on this study population. Therefore, only the internal validity of using the three items was examined in the present study, and further studies on external validity are needed.

In this study, we examined the accuracy of screening for excess salt intake using ROC analysis in addition to relative correlation analysis, thereby quantifying the degree of error. The simplified questionnaire, salt check sheet, was found to be useful in screening for excessive salt intake, allowing measurement error, only in males, and the total salt check sheet score of 13 points corresponded to the Japanese average daily salt intake of 10 g. Therefore, the salt check sheet has the potential to be used as a simple screening tool for assessing individuals’ salt intake for males, particularly in settings such as health check-ups and nutrition education programs. However, excluding participants on any medication slightly improved the correlation among males; therefore, caution may be necessary when applying this to patients on medication. On the other hand, for females, it was suggested that it could be used only on the basis of three specific items: “Frequency of eating miso soup, soup, and so on,” “How much soup in noodles, such as udon, ramen, or others, do you consume?”, and “Do you think you eat a lot?”, and the score of 4 points corresponded to 10 g of daily salt intake, although the salt sheet was not found to be useful when using the total score. The external validity of screening based on these three specific items requires further investigation.
